# Simultaneous serological assessment of four zoonotic rickettsiae among dogs near the United States-Mexico border

**DOI:** 10.1371/journal.pntd.0014099

**Published:** 2026-03-16

**Authors:** Francesca Rubino, Sarah A. Hamer, Andres M. López-Pérez, Samantha Schuchman, Kailyn Lozano, Alexandra Saffold, Mario A. Rodríguez-Pérez, Nadia A. Fernández-Santos, Janet Foley

**Affiliations:** 1 Department of Medicine and Epidemiology, School of Veterinary Medicine, University of California, Davis, California, United States of America; 2 Department of Veterinary Integrative Biosciences, College of Veterinary Medicine & Biomedical Sciences, Texas A&M University, College Station, Texas, United States of America; 3 Red de Biología y Conservación de Vertebrados, Instituto de Ecología, A.C., Xalapa, México; 4 Instituto Politécnico Nacional, Centro de Biotecnología Genómica, Reynosa, Tamaulipas, México; 5 Department of Entomology, Texas A&M AgriLife Research, Weslaco, Texas, United States of America; Postgraduate Institute of Medical Education and Research, INDIA

## Abstract

Obligately intracellular rickettsiae cause a broad spectrum of disease in humans and animals, ranging from mild illness to life-threatening infections. Multiple species co-circulate along the southern United States of America–northern Mexico border, yet their seroprevalence in susceptible hosts remains incompletely understood. Dogs serve as key amplifying hosts for several of these pathogens, including *Rickettsia rickettsii* and *Rickettsia massiliae*, and have been shown to be infected by flea-borne *Rickettsia typhi* and *Rickettsia felis*. To better characterize exposure and potential co-infection patterns, we conducted a large binational seroepidemiologic study of 779 dogs from urban households and shelter settings across seven subregions along both sides of the border. Using a custom multiplex micro-immunofluorescence assay, we simultaneously screened for antibodies to *R. rickettsii*, *R. massiliae*, *R. typhi*, and *R. felis*. Overall, 41.2% of dogs were seroreactive to at least one pathogen, with the highest seroprevalence for *R. felis* (19.3%), followed by *R. massiliae* (15.7%), *R. typhi* (14.5%), and *R. rickettsii* (9.8%). Co-seroreactivity was common, particularly between *R. typhi* and *R. felis*, with 34.2% of *R. typhi*–seroreactive samples also seroreactive to *R. felis*, and 16.7% exhibiting high titers to both. In contrast, *R. rickettsii* and *R. massiliae* showed limited overlap (15.2% co-seroreactive; 6.4% with high titers), suggesting possible cross-protection or competitive exclusion. Spatial analyses revealed distinct geographic patterns: *R. massiliae* predominated in western Baja California, *R. rickettsii* was concentrated in Mexicali and the Rio Grande Valley, and *R. felis* was widely distributed. Seroreactivity patterns were generally consistent across age and sex but varied modestly between shelter and neighborhood dogs, particularly for *R. typhi*. These findings highlight the complex ecology of rickettsial pathogens in a binational context, underscore the importance of dogs as sentinels for human risk, and provide a foundation for future studies on vector-host-pathogen interactions, cross-protective immunity, and spatial epidemiological risk.

## Author summary

Rickettsial diseases are caused by bacteria spread by ticks, fleas, and lice and can range from mild illness to life-threatening infections. Along the border between the United States of America and Mexico, several rickettsial species—including *Rickettsia rickettsii*, *Rickettsia massiliae*, *Rickettsia typhi*, and *Rickettsia felis*—infect both people and dogs. However, traditional tests often cannot accurately distinguish among these closely related bacteria, and antibodies to one species can cross-react with others, making it difficult to determine which pathogens are present in different regions or how common exposure is among hosts. To address this, we tested blood samples from nearly 800 dogs using an assay that is capable of detecting antibodies to the four pathogens at once. We found that exposure to these bacteria was common but varied by region. Our results highlight the complex ecology of rickettsial transmission and emphasize the value of dogs as indicators of human disease risk.

## Introduction

Obligate intracellular rickettsiae span a wide pathogenic and ecological spectrum across geography, arthropod vectors, and hosts, causing mild infections to life-threatening disease. Five rickettsial species—*Rickettsia rickettsii*, *Rickettsia massiliae*, *Rickettsia parkeri, Rickettsia typhi*, and *Rickettsia felis*—co-circulate along the border between the southern United States of America (USA) and northern Mexico (our region of focus, RoF) [1–4]. Understanding their seroprevalence in susceptible hosts is a critical step toward unraveling the complex ecological and immunological interactions among them.

The most virulent of these rickettsiae, *R. rickettsii*, has caused deadly outbreaks of Rocky Mountain spotted fever (RMSF) in people and dogs throughout the RoF, with up to 80% of reported cases resulting in death in some locations [3]. Although transmitted by several tick species, the brown dog tick (*Rhipicephalus sanguineus* sensu lato) is the principal vector in the RoF, where dogs serve as amplifying hosts. *Rickettsia massiliae*, also transmitted by brown dog ticks, has recently been identified in North America [2]. Although associated with disease in both humans and dogs, its full pathogenic potential remains poorly understood.

In contrast, *R. typhi* is flea-borne and historically associated with rodent reservoirs [5], though research shows that opossums (*Didelphis virginiana*) are important host species as well [6]. Additionally, infected dogs have been identified in Texas, USA and Yucatan, Mexico [7,8], and infection has been confirmed based on seroreactivity, polymerase chain reaction (PCR) positivity, and direct culture [9]. Furthermore, *R. typhi* was recently detected in brown dog ticks by PCR and restriction fragment length polymorphism, and confirmed via DNA sequencing of fragments obtained from isolates [9]. Dogs have been implicated as possible hosts for another flea-borne pathogen, *R. felis,* with PCR-confirmed infections [10,11]. Infected dogs can experience prolonged rickettsiemia and transmit *R. felis* to fleas [12]. Still, the relative importance of dogs in sustaining transmission cycles for both *R. typhi* and *R. felis* remains poorly understood.

Interactions among rickettsial species within a community may influence host susceptibility, immune response, patterns of transmission, interpretation of diagnostic assays, and control strategies. For example, hosts that are infected with one species may experience partial cross-protective immunity against other species, while vectors with pre-existing rickettsial infection may resist superinfection with other rickettsiae [13–17]. However, resolving the impacts of such interactions remains difficult in part due to the limited resolution of existing diagnostic tools. PCR-based approaches, while species-specific, often fail to detect pathogen DNA due to transient and low rickettsemia in hosts [18]. Serology, used to diagnose rickettsial infections for over a century, is more sensitive for detecting past exposure than PCR, but cross-reactivity among rickettsial antigens limits its ability to identify which pathogens circulate in a region. Therefore, more refined serologic strategies, such as multiplex testing that evaluates multiple pathogens simultaneously, may help reveal broad patterns of exposure and guide hypotheses about potential cross-reactivity, coinfection, and spatial variation in risk. When paired with spatial data, this approach allows for more nuanced interpretation of exposure patterns, regional trends, and co-seroreactivity.

To address important gaps in knowledge, we conducted a seroepidemiologic study estimating the relative seroprevalence of antibodies to *R. rickettsii*, *R. massiliae*, *R. typhi*, and *R. felis* in dogs from communities along both sides of the USA–Mexico border. Using a custom-designed multiplex serological assay, we simultaneously screened for exposure to all four pathogens, enabling more refined comparisons than single-pathogen testing allows. We analyzed patterns across space and demographics, identifying how seroreactivity varied within individuals, populations, and the RoF. Our findings shed light on the complex ecology of rickettsial transmission in this binational region and lay the groundwork for future studies on reservoir competence, vector-host-pathogen interactions, and spatial epidemiological risk.

## Methods

### Ethics statement

This study was a secondary analysis of samples collected in previous studies. No new sampling was conducted, and all analyses were performed in accordance with the original approvals. Animal sampling protocols for each location were originally approved by the following Institutional Animal Care and Use Committees (IACUCs) or affiliated groups:

- **Academic Group of Animal Health, Instituto de Investigaciones en Ciencias Veterinarias, Universidad Autónoma de Baja California (UABC)**: Mexicali and Tijuana
- **Academic Group for Diagnosis of Infectious Diseases, Instituto de Investigaciones en Ciencias Veterinarias, UABC**: Mexicali
- **Texas A&M University IACUC**: Reynosa and Rio Grande
- **University of California, Davis IACUC**: Ensenada, Rosarito, Tecate, Imperial County, San Diego County, Mexicali, and Tijuana

### Sample sources and collection methods

Archived canine blood samples from prior epidemiological studies and animal shelters within our RoF were obtained from the cities and surrounding areas of Ensenada, Mexicali, Rosarito, Tecate, and Tijuana (Baja California state, Mexico); the city of Reynosa (Tamaulipas state, Mexico); Imperial County and San Diego County (California state, USA); and the Rio Grande Valley region (Texas state, USA) [19–25]. In total, 779 dogs were analyzed.

Whole blood samples from neighborhood dogs in Mexicali in August and September 2017, Tijuana in May 2021, and Ensenada in October 2022 were collected using a randomized neighborhood-based sampling design [21,24,25]. Neighborhoods were selected based on the recent occurrence of RMSF cases, along with randomly selected control neighborhoods. Twenty neighborhoods were sampled in Ensenada and Mexicali, while 15 neighborhoods were sampled in Tijuana. Within each neighborhood, a city block was randomly selected, and five households per block were surveyed using a counterclockwise approach. Up to five dogs per household were sampled via venipuncture into EDTA tubes. Dogs that could not be safely restrained or for which owner consent was not obtained were excluded. A total of 174 samples from dogs in Ensenada, 163 in Tijuana, and 215 in Mexicali were available. From each site, 100 samples were selected using a random sampling approach designed to reduce clustering. All samples at each study site were assigned a random number. To limit intra-household correlation and reduce overrepresentation of individual city blocks, only the first two samples from different households were included.

Plasma samples from Reynosa were obtained from dogs sampled in six disadvantaged neighborhoods between April and August 2019, selected based on low or low-medium socioeconomic status. These dogs were previously assessed for infection with several other vector-borne pathogens [19,23]. Dogs were enrolled through visits to homes, vacant lots, and door-to-door recruitment. Free rabies vaccinations were offered as an incentive for participation.

Serum samples from Rio Grande Valley of south Texas were obtained from dogs residing in ‘colonias’ in 2017–2019 using a community recruitment approach as presented previously [22], with free rabies vaccinations offered as incentives for enrollment.

A total of 283 samples were obtained from dogs housed in animal shelters and veterinary clinics. Of these, 239 were part of a study on shelter dogs conducted between October 2021 and May 2022 from Bonita (a census-designated place) and the city of Carlsbad in San Diego County; the city of El Centro in Imperial County; and the cities of Mexicali, Rosarito, Tecate, and Tijuana [20]. These dogs were selected from a convenience sample of dogs aged ≥6 weeks who were not receiving medical treatment. An additional 44 samples were contributed by clinics participating in the Border Tick Rickettsia Surveillance Program (BiTeRS), including those from Imperial County (March and October 2023; July and August 2024) and San Diego County (October, July, and December 2022 July 2022). The data used in this study are available in the Dryad Digital Repository [26].

### Serological testing

To address the challenges of serological cross-reactivity and improve differentiation among co-circulating rickettsial pathogens, we employed a custom-designed multiplex micro-immunofluorescence assay (MIF) that simultaneously detects antibodies against *R. rickettsii*, *R. massiliae*, *R. typhi*, and *R. felis* created by Fuller Laboratories (now VMRD Inc.; Fullerton, California). Each well contained four distinct spots coated with cells infected with *R. rickettsii*, *R. massiliae*, *R. typhi*, and *R. felis*. Samples consisted of canine whole blood, serum, or plasma and were stored in the laboratory at −70°C until testing. At the time of testing, samples were thawed and diluted 64 and 128-fold for initial screening in phosphate-buffered saline (PBS). Twenty-five µL of dilute sample were applied to each slide well and incubated at 37°C in a humidified chamber for 20 minutes. Slides were then washed three times with PBS. A 10-fold dilution of fluorescein isothiocyanate-conjugated anti-dog IgG heavy and light chain antibodies (LGC SeraCare, Milford, MA) in PBS was applied, and the slides were incubated again for 20 min at 37°C. After three additional PBS washes, Eriochrome Black (Sigma- Aldrich, St. Louis, MO) was added as a counterstain during the final wash. A 10% glycerol solution (pH 7.4) was added to each well, and coverslips were applied. Slides were examined in the Foley Lab by a single trained reader using a UV fluorescence microscope. PBS was included as a negative control, and serum from dogs previously confirmed to be seropositive served as positive controls. Samples were considered positively seroreactive if the titer was ≥ 64 with bright green fluorescence observed in a characteristic pattern. If a sample tested positive for antibodies to any pathogen on the first two dilutions, it was retested at 1:256 and 1:512 dilutions. Titers of 64 and 128 were scored as low seroreactivity, and titers of 256 and 512 scored as high seroreactivity.

### Statistical analysis

We examined patterns of co-seroreactivity, geographic distribution, and demographic variation in our data. Dogs were considered co-seroreactive if they exhibited titers to both of a given pair of pathogens. For each pair, we calculated the proportion of seroreactive individuals to one pathogen who were also seroreactive to the second, and then averaged the two proportions to obtain a symmetric co-seroreactivity value: $\frac{\left(\left(n_{AB}{/n}_{A}\right)+\left(n_{AB}/n_{B}\right)\right)}{2}$. This measure represents the average likelihood that a dog seroreactive to one pathogen is also seroreactive to the other, relative to each pathogen’s seroprevalence.

To further understand co-seroreactivity patterns, we employed non-metric multidimensional scaling (NMDS). Individual dogs’ seroreactivity profiles, i.e., the pattern of serological titers measured against the four rickettsial pathogens, were log₂-transformed, and Bray-Curtis dissimilarity values were calculated to quantify differences among dogs. This dissimilarity metric captures variation in both the pathogens to which a dog is seroreactive and the magnitude of those responses (e.g., whether dogs with high titers to *R. rickettsii* also have high titers to *R. massiliae*, and are thus more likely co-infected). Log₂-transforming the variables standardized the titers for analysis and ensured that each doubling step contributed equally to the dissimilarity, preventing rare high titers from disproportionately influencing the results. NMDS was then used to reduce these dissimilarities into two dimensions, enabling visualization of similarity patterns among individual dogs’ serologic profiles and assessment of co-reactivity among antibodies to different pathogens within dogs.

To ensure a robust and conservative classification of likely exposures based on antibody responses amid potential cross-reactivity in our spatial and demographic analyses that follow, we applied the following approach. Previous studies suggest that the serological titer associated with the true pathogen to which a host is exposed may be two- to four-fold higher than it is for other cross-reactive rickettsiae [27–30]. In our analysis, dogs who were co-seroreactive were classified as having been exposed to a single pathogen if they exhibited a ≥ 4-fold higher titer to that antigen relative to other pathogens on the slide. Those with multiple positive titers but no clear ≥4-fold difference were not assigned to a pathogen. To account for possible titer fluctuations and true coinfection, we conducted parallel analyses using both the full dataset (including ambiguous or multiple-pathogen responses) and a more specific subset limited to seronegative dogs and those with clearly attributable seroreactivity (i.e., reactivity to a single pathogen or a ≥ 4-fold higher titer to one pathogen relative to others).

We characterized spatial patterns of seroreactivity to each pathogen using three complementary approaches. Dogs were grouped into seven geographic subregions based on sampling location (S1 Fig):

- Ensenada: Neighborhood study in Ensenada, Mexico (n = 96)
- Tijuana metropolitan area: Neighborhood study and shelters in Tijuana, Tecate, and Rosarito, Mexico (n = 177)
- San Diego County: Shelters and veterinary clinics in California from Alpine, Bonita, Boulevard, Campo, Carlsbad, El Cajon, and San Diego, California (n = 92)
- Imperial County: Shelters and veterinary clinics in Imperial County, CA (n = 48)
- Mexicali: Neighborhood study and shelters in Mexicali, Mexico (n = 166)
- Mexico–Texas (MX-TX) border: Reynosa, Mexico and the Rio Grande Valley, Texas (n = 200)

First, we assessed whether individual dogs’ seroreactivity profiles varied across subregions by applying a Permutational Multivariate Analysis of Variance (PERMANOVA) on the Bray-Curtis dissimilarity matrix. Second, we mapped the attributable seroprevalence to each pathogen in each subregion. This was done by using pie charts made in ArcGIS (3.5) and 95% confidence intervals were calculated using the Wilson score method. Third, we created bar plots for each subregion showing the proportion of seroreactive dogs attributable to each pathogen.

Age (n = 390) and sex (n = 392) data were available from neighborhood sampling in Ensenada, Mexicali, and Tijuana, and from the shelter- and clinic-based sampling. In Tijuana and Mexicali, samples were available from dogs in both neighborhoods (n = 200) and animal shelters (n = 126). Comparisons across age group (<1 year vs. ≥ 1 year), sex, or source (neighborhood vs. shelter) were made using Fisher’s exact test. Statistical significance was set at α = 0.05. All analyses were conducted using R (version 4.3.2).

## Results

Overall, 41.2% of dogs (321/779) were seroreactive to at least one of the four rickettsial pathogens. The most commonly detected antibodies (Table 1) were against *R. felis* (19.3%), followed by *R. massiliae* (15.7%), *R. typhi* (14.5%), and *R. rickettsii* (9.8%). The median titer was the same, 128, for all four pathogens. Two hundred and eleven dogs exhibited seroreactivity to only one pathogen and 110 dogs exhibited seroreactivity to two or more pathogens.

**Table 1:** Distribution of antibody titers for each *Rickettsia* species.

Pathogen	Titer	Total % (n = 779)	Attributable % (n = 680)
*R. rickettsii*	All	9.8 (76)	6.2 (42)
	64	0.1 (1)	0.0 (0)
	128	6.9 (54)	4.6 (31)
	256	1.7 (13)	0.9 (6)
	512	1.0 (8)	0.7 (5)
*R. massiliae*	All	15.7 (122)	8.8 (60)
	64	0.3 (2)	0.0 (0)
	128	10.0 (78)	6.0 (41)
	256	2.8 (22)	1.5 (10)
	512	2.6 (20)	1.3 (9)
*R. typhi*	All	14.5 (113)	6.8 (46)
	64	1.3 (10)	0.6 (4)
	128	9.0 (70)	4.1 (28)
	256	2.3 (18)	0.9 (6)
	512	1.9 (15)	1.2 (8)
*R. felis*	All	19.3 (150)	10.9 (74)
	64	0.8 (6)	0.2 (1)
	128	11.9 (93)	6.0 (41)
	256	1.8 (14)	1.0 (7)
	512	4.8 (37)	3.7 (25)
Grand Total	All	41.2 (321)	32.7 (222)

“Total seroreactive” includes all dogs with detectable antibodies to a given pathogen; “attributable seroreactive” is limited to dogs whose serostatus could be attributed to a single pathogen or classified as seronegative. Percentages represent the proportion within the respective group, counts are in parentheses.

### Patterns of co-seroreactivity

Dogs exhibited co-seroreactivity to all combinations of pathogens (S1 Table). For all pathogen pairs except for some combinations with *R. rickettsii,* there were dogs where titers as high as 512 were observed for both pathogens in the pair. When dogs were seroreactive against *R. rickettsii* and either *R. massiliae* or *R. typhi*, one pathogen or the other had a higher titer. Five dogs were seroreactive to all four pathogens (1 from Ensenada and 4 from Reynosa — the two subregions with the highest overall seroprevalences) and 19 dogs were seroreactive to at least 3 pathogens (11 from Ensenada, 4 from Rio Grande Valley, 3 from Reynosa, and 1 from Imperial County). After accounting for the relative frequency of seroreactivity to each pathogen using symmetric averaging, overall dogs were most frequently co-seroreactive to *R. typhi* and *R. felis* (34.2% for all titers ≥64, 16.7% for high titers ≥256) and least frequently co-seroreactive to *R. rickettsii* and *R. massiliae* (15.2% for all titers, 6.4% for high titers) (S2 Table). Among high-titer responses, the most common co-seroreactivity patterns were *R. massiliae* with *R. felis* (25.8%) or with *R. typhi* (24.0%).

Among the 110 dogs seroreactive to multiple pathogens, 11 exhibited a ≥ 4-fold higher antibody titer to a single pathogen (5 to *R. felis,* 4 to *R. massiliae*, 1 to *R. typhi*, and 1 to *R. rickettsii*). Based on these criteria, 680 dogs (87.3%) were included in the attributable analyses (i.e., dogs that were seronegative, reactive in only one slide well, or reactive to a single pathogen with a ≥ 4-fold higher titer compared to others).

Non-metric multidimensional scaling (NMDS) effectively summarized the individual differences in dogs’ seroreactivity profiles into two dimensions, with a good overall fit (stress = 0.07). Seroreactivity to *R. rickettsii* and *R. massiliae s*howed significant opposing orientations along the first NMDS axis (S2 Fig), suggesting that dogs exposed to one of these pathogens were less likely to be exposed to the other. Despite substantial co-seroreactivity, *R. typhi* and *R. felis* displayed opposing orientations along the second NMDS axis, indicating an inverse relationship in relative titers: dogs with higher titers to one pathogen tended to have lower titers to the other within their overall seroreactivity profile. PERMANOVA confirmed that dogs’ seroreactivity profiles differed significantly between subregions (F = 2.95, p = 0.001; R² = 0.045), but estimated that only 5% of the variation was explained by location.

### Patterns across space

**Fig 1:**
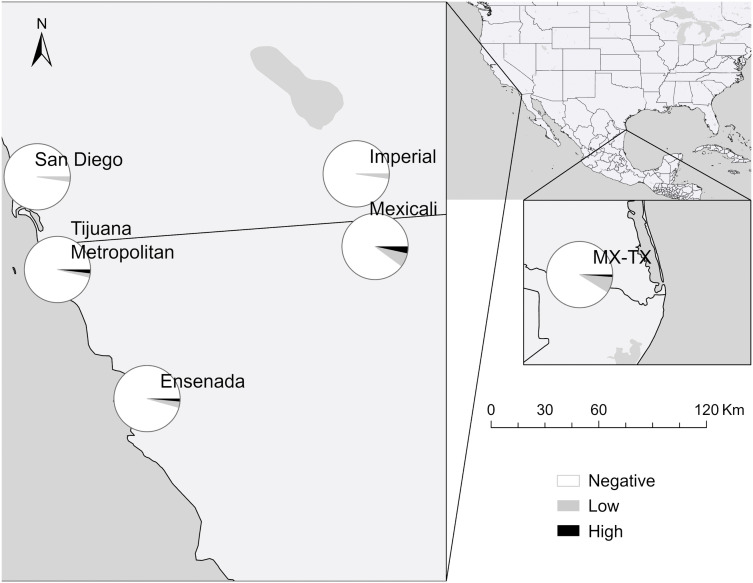
Seroprevalence of antibody titers against *Rickettsia rickettsii* in dogs across region of focus by titer level. Titers are: Negative, Low (64–128), and High (256–512). Map created using ArcGIS software. Base layers made with Natural Earth: https://www.naturalearthdata.com/downloads/. Natural Earth data are in the public domain; terms of use: https://www.naturalearthdata.com/about/terms-of-use/.

**Fig 2:**
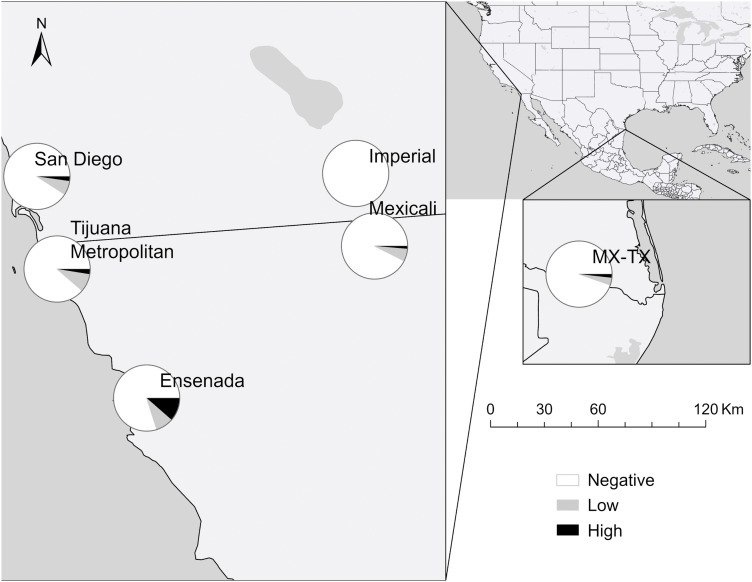
Seroprevalence of antibody titers against *Rickettsia massiliae* in dogs across the region of focus by titer level. Titers are: Negative, Low (64–128), and High (256–512). Map created using ArcGIS software. Base layers made with Natural Earth: https://www.naturalearthdata.com/downloads/. Natural Earth data are in the public domain; terms of use: https://www.naturalearthdata.com/about/terms-of-use/.

The inverse relationship between *R. rickettsii* and *R. massiliae* that was evident in the NMDS ordination was also apparent in their contrasting east-west geographic distributions as depicted in the maps. *Rickettsia rickettsii* seroprevalence was highest in the east, peaking at 13.2% among dogs in Mexicali shelters and 11.5% in the Rio Grande Valley of the Mexico–Texas subregion (Fig 1), while sites further west had rates at 5.3% and lower. Conversely, *R. massiliae* seroprevalence was highest in the west (20.0% in Ensenada; Fig 2), and lower in the east, with 5.3–7.4% in Mexicali and the Mexico–Texas subregion. A north–south gradient was also evident, distinguishing parts of southern California from northern Mexico. While all subregions in Mexico showed reactivity to both pathogens, California had the lowest *R. rickettsii* rates in the region (2.2–2.3%) and no high titers. Antibodies to *R. massiliae* similarly were not detected in Imperial County, although it was present in San Diego at 9.1%, nearing levels observed in the Tijuana metropolitan area (11.3%). Detailed results with 95% confidence intervals are provided in Table 2, with titer-specific breakdowns in S3 Table.

**Table 2:** Seroprevalence of reactivity expressed as antibody titers ≥64 to each pathogen by subregion and study site.

Subregion/ Study Site	Any Pathogen % (n) all; attributable	*R. rickettsii* % (n) all; attributable	*R. massiliae* % (n) all; attributable	*R. typhi* % (n) all; attributable	*R. felis* % (n) all; attributable
**Ensenada** **(96; 70)**	**61.5 (59);** **47.1 (33)**	**9.4 (9);** **4.3 (3)**	**38.5 (37); 20.0 (14)**	**27.1 (26); 10.0 (7)**	**32.3 (31); 12.9 (9)**
**Tijuana metropolitan area (177; 159)**	**35.0 (62);** **27.7 (44)**	**7.3 (13);** **3.8 (6)**	**15.8 (28); 11.3 (18)**	**11.3 (20);** **6.3 (10)**	**10.7 (19);** **6.3 (10)**
Tijuana N. (100; 94)	32.0 (32); 27.7 (26)	7.0 (7); 5.3 (5)	12.0 (12); 10.6 (10)	8.0 (8); 5.3 (5)	11.0 (11); 6.4 (6)
Tijuana S. (60; 50)	38.3 (23); 26.0 (13)	6.7 (4); 2.0 (1)	16.7 (10); 6.0 (3)	18.3 (11); 10.0 (5)	13.3 (8); 8.0 (4)
**San Diego** **(92; 88)**	**31.5 (29);** **28.4 (25)**	**3.3 (3);** **2.3 (2)**	**13.0 (12);** **9.1 (8)**	**5.4 (5);** **5.7 (5)**	**15.2 (14); 11.4 (10)**
**Imperial** **(48; 45)**	**20.8 (10);** **15.6 (7)**	**2.1 (1);** **2.2 (1)**	**2.1 (1);** **0.0 (0)**	**10.4 (5);** **4.4 (2)**	**14.6 (7);** **8.9 (4)**
**Mexicali** **(166; 149)**	**40.4 (67);** **33.6 (50)**	**12.1 (20); 10.1 (15)**	**12.1 (20);** **7.4 (11)**	**12.1 (20);** **6.0 (9)**	**16.3 (27); 10.1 (15)**
Mexicali N. (100; 96)	36.0 (36); 33.3 (32)	10.0 (10); 8.3 (8)	12.0 (12); 9.4 (9)	5.0 (5); 4.2 (4)	14.0 (14); 11.5 (11)
Mexicali S. (66; 53)	47.0 (31); 34.0 (18)	15.2 (10); 13.2 (7)	12.1 (8); 3.8 (2)	22.7 (15); 9.4 (5)	19.7 (13); 7.6 (4)
**MX-TX** **(200; 169)**	**46.5 (94);** **37.3 (63)**	**15.0 (30);** **8.9 (15)**	**12.0 (24);** **5.3 (9)**	**18.5 (37);** **7.7 (13)**	**26.0 (52); 15.4 (26)**
Reynosa (100; 82)	52.0 (52); 41.5 (34)	14.0 (14); 6.1 (5)	18.0 (18); 6.1 (5)	20.0 (20); 8.5 (7)	31.0 (31); 20.7 (17)
Rio Grande Valley (100; 87)	42.0 (42); 33.3 (29)	16.0 (16); 11.5 (10)	6.0 (6); 4.6 (4)	17.0 (17); 6.9 (6)	21.0 (21); 10.3 (9)
Grand Total (779; 680)	41.2 (321); 32.7 (222)	9.8 (76); 6.2 (42)	15.7 (122); 8.8 (60)	14.5 (113); 6.8 (46)	19.3 (150); 10.9 (74)

Values show the percentage seropositive with raw counts in parentheses. For each location, two values are provided: one for all samples, and one for attributable samples, which include only dogs whose serostatus could be attributed to a single pathogen or classified as seronegative. Subregions are bolded, with associated study sites listed below. Sites with ≤50 samples are excluded individually but included in subregional totals where applicable. Neighborhoods are marked with an “N” and shelters with an “S”.

**Fig 3:**
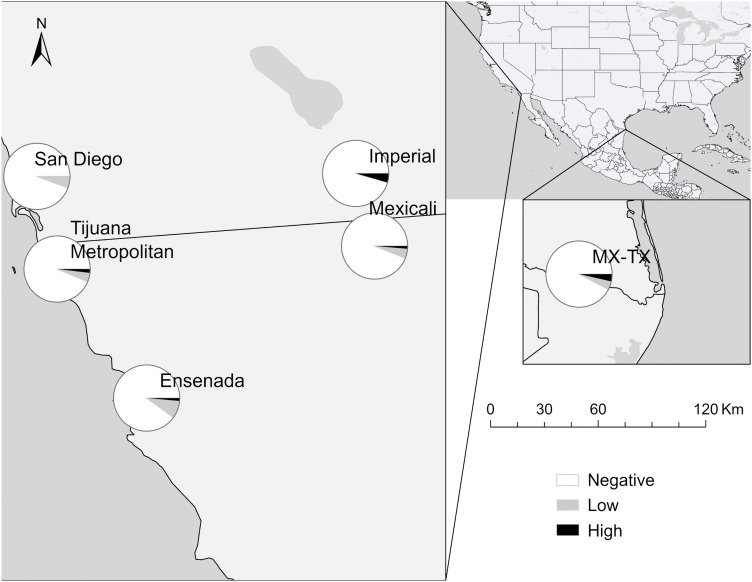
Seroprevalence of antibody titers against *Rickettsia typhi* in dogs across the region of focus by titer level. Titers are: Negative, Low (64–128), and High (256–512). Map created using ArcGIS software. Base layers made with Natural Earth: https://www.naturalearthdata.com/downloads/. Natural Earth data are in the public domain; terms of use: https://www.naturalearthdata.com/about/terms-of-use/.

**Fig 4:**
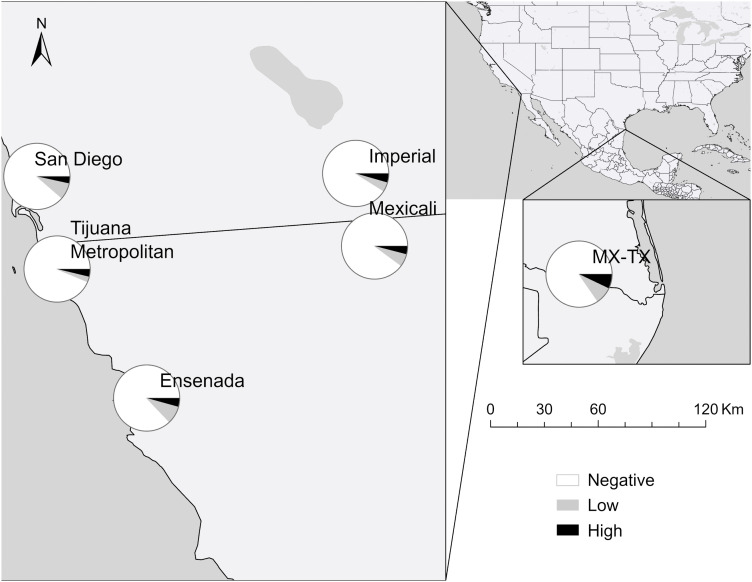
Seroprevalence of antibody titers against *Rickettsia felis* in dogs across the region of focus by titer level. Titers are: Negative, Low (64–128), and High (256–512). Map created using ArcGIS software. Base layers made with Natural Earth: https://www.naturalearthdata.com/downloads/. Natural Earth data are in the public domain; terms of use: https://www.naturalearthdata.com/about/terms-of-use/.

**Fig 5:**
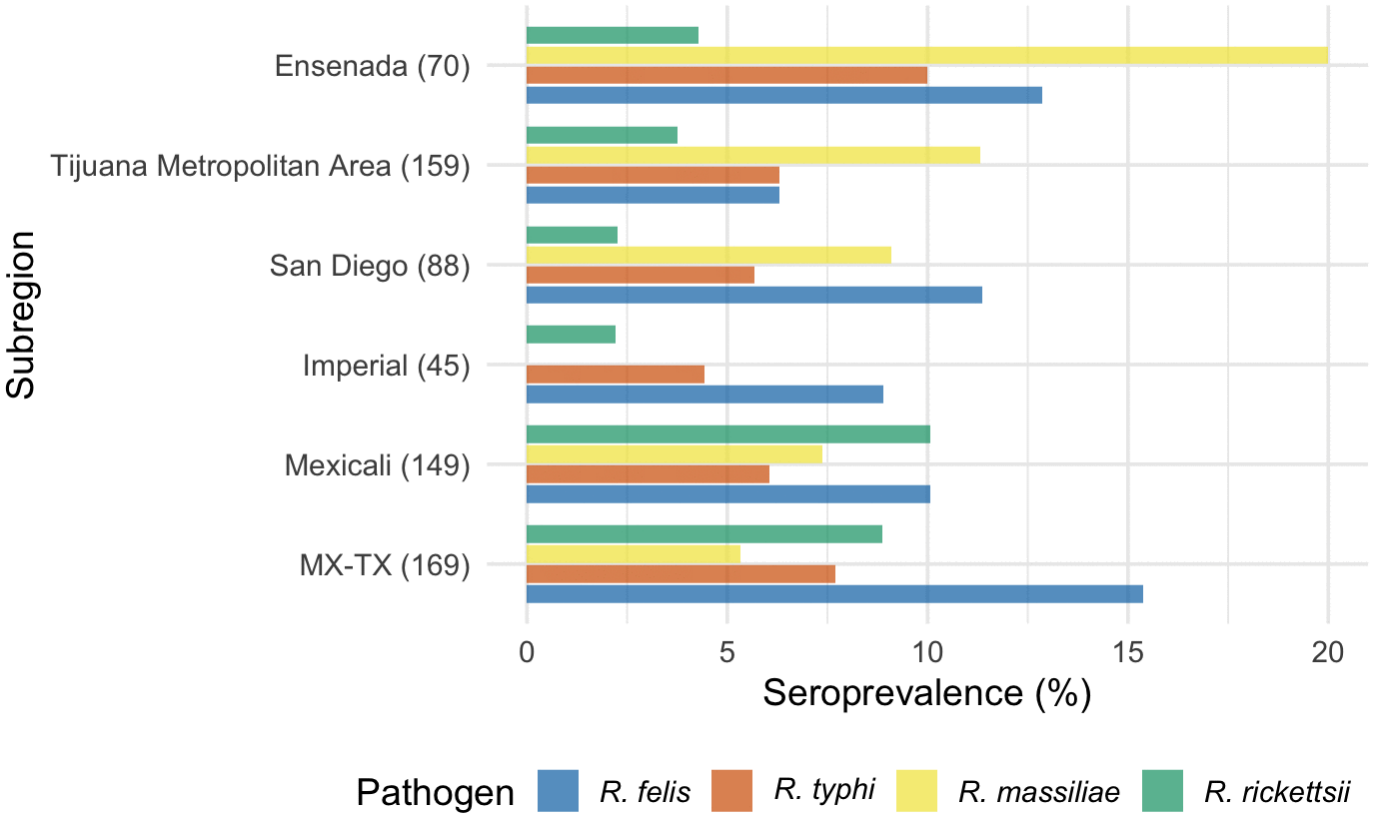
Percentage of dogs seroreactive to specific Rickettsia species by subregion. Sample sizes are shown in parentheses and include only dogs with serostatus attributed to a single pathogen or classified as seronegative.

The inverse relationship between *R. typhi* (Fig 3) and *R. felis* (Fig 4) detected by the NMDS ordination was less geographically distinct. *Rickettsia typhi* had a north–south gradient with prevalence peaking in the south, reaching 10.0% in Ensenada, and similarly high levels in Reynosa (8.5%). The lowest levels were observed in the north, ranging between 4–6% in Mexicali, Imperial, San Diego, and among neighborhood dogs in Tijuana. *Rickettsia felis*, on the other hand, was observed at similar levels of 10–13% throughout much of the RoF. A hotspot was detected in Reynosa (20.7%), with levels falling to half that in the neighboring Rio Grande Valley (10.3%). The lowest levels were observed in the Tijuana metropolitan area (6.3%). These subregional patterns are visualized in Fig 5.

**Fig 6:**
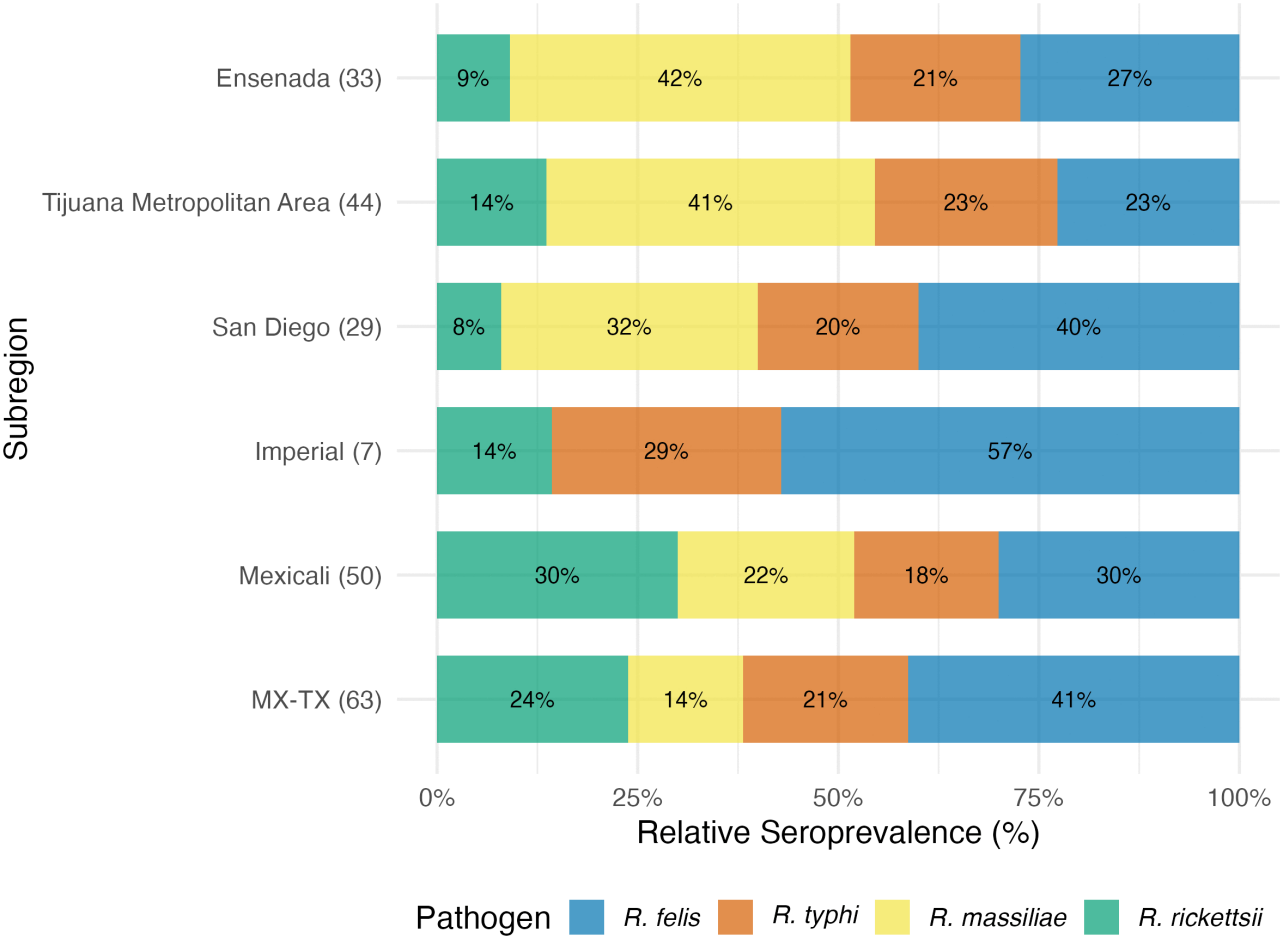
Relative percentage of dogs seroreactive to each Rickettsia species by subregion. Number of positive dogs at each site are shown in parentheses and include only dogs with serostatus attributed to a single pathogen or classified as seronegative.

The relative proportions of each pathogen among seroreactive dogs by subregion revealed which regions were most affected by which pathogen, forming three distinct spatial patterns across the RoF: 1) *R. massiliae* accounted for the majority of exposures in the far-west on the Mexico side, 2) *R. felis* led exposures in California; and 3) *R. rickettsii* intensified from Mexicali eastward (Fig 6). These patterns held for both the total sample and the subset of attributable exposures (Table 2). Among subregions, Ensenada had the highest overall seroreactivity with 61.5% (59/96) of dogs seroreactive to at least one pathogen. Seroreactivity was reduced by about half in the Tijuana metropolitan area (62/177, 35.0%) to similar levels seen in the other subregions. Regardless, in both places the most common pathogen among seropositive dogs was *R. massiliae*, accounting for 42.4% of positives in Ensenada and 40.9% in the Tijuana metropolitan area. This was followed by *R. felis* (27.3% and 22.7%, respectively), *R. typhi* (21.2% and 22.7%), and *R. rickettsii* (9.1% and 13.6%).

Across the border, overall seroprevalences in San Diego (33.8%, 23/68) and Imperial Counties (27.1%, 13/48) were comparable to levels observed in the Tijuana metropolitan area. In contrast, *R. felis* was the most prevalent pathogen among seropositive dogs in both California counties (40.0% in San Diego and 57.1% in Imperial). In San Diego, *R. massiliae* followed at 32.0%, but it was absent in Imperial County—which had a relatively small number of seropositive dogs (n = 7). *Rickettsia typhi* was similarly detected in both counties (20.0% in San Diego and 28.6% in Imperial), while *R. rickettsii* was least common in San Diego (8.0%) and remained relatively low in Imperial (14.3%).

Overall seroprevalence was similar in Mexicali (34.0%) and along the Mexico–Texas border (37.3%). While *R. felis* still accounted for much of the seroreactivity (30.0% in Mexicali, 50.0% in Reynosa, and 31.0% in the Rio Grande Valley), *R. rickettsii* was exceptionally high. In Mexicali, *R. rickettsii* comprised an equal proportion of seroreactive dogs, and in the Rio Grande Valley an even greater proportion (34.5%), though it dropped to just 14.7% in Reynosa (averaging 23.8% across the MX-TX border sites). *Rickettsia massiliae* and *R. typhi* occurred at similar levels in Mexicali (22.0% and 18.0%, respectively), while at the border, *R. typhi* was more common (20.6%) and *R. massiliae* less so (14.3%).

### Patterns across demographic classes

Most seroprevalence estimates stratified by sex and age differed by less than 3 percentage points (S4 Table). The only exception was *R. felis*, for which dogs older than 1 year had over twice the seroprevalence of younger dogs, though the difference was not statistically significant (p = 0.231). These demographic patterns were consistent in the total dataset and the subset of attributable samples. The percentage of dogs who were seroreactive to multiple pathogens (S5 Table) was also consistent across demographic groups.

Greater variation was observed in seroreactivity among sheltered vs. neighborhood dogs, particularly for *R. typhi* (S6 Table). In the total sample, 47.0% of shelter dogs and 36.0% of neighborhood dogs in Mexicali, and 38.3% of shelter dogs and 32.0% of neighborhood dogs in Tijuana, were seroreactive to any pathogen. These differences were not statistically significant (p = 0.196 and p = 0.492, respectively). Shelter dogs in Mexicali were significantly more likely to be seroreactive to *R. typhi* than neighborhood dogs in the total sample (OR = 5.53, 95% CI: 1.78–20.58, p = 0.001). However, a similar pattern was observed for co-seroreactivity, with an identical odds ratio and confidence interval. A similar, though not statistically significant, trend was observed in Tijuana (OR = 2.57, 95% CI: 0.87–7.87, p = 0.075). As a result, when restricting the analysis to the subset of dogs with attributable exposures, no statistically significant differences in *R. typhi* or overall seroreactivity were observed between the two groups. For the other three pathogens, seroreactivity rates were comparable between shelter and neighborhood dogs.

## Discussion

*Rickettsia rickettsii, R. massiliae, R. typhi,* and *R. felis* cause a broad range of symptoms and clinical signs, interact in complex ways within vectors and hosts, and often cross-react in serological assays, complicating diagnosis and surveillance. Nevertheless, understanding patterns of exposure and spatial distribution in dogs is a critical step toward unraveling the complex ecological and immunological interactions among *Rickettsia* species. This study presents the first simultaneous serological screening for all four species in a large, binational cohort of dogs. Exposure to at least one of the four pathogens was common (41.2%), but with marked geographic variation in both seroprevalence and antibody titers, suggesting distinct ecological or transmission dynamics across the RoF.

Even for acute infections, attributing a specific rickettsial pathogen to a clinically ill or seroreactive person or dog is challenged by non-specific clinical signs or undetectable parasite in DNA in blood or accessible tissue for diagnostic PCR. In both humans and dogs, serological diagnosis primarily relies on IFA, considered the gold standard. Results are typically interpreted using a titer cutoff, often 64, to distinguish negative from positive tests [31]. Variation in antibody titers may arise from individual immune differences, timing of the most recent exposure, repeated exposures, or co-infections. For example, titer dynamics in people are not well-documented, but in dogs, IgG against *R. rickettsii* was first detectable 22–28 days after exposure, peaked at day 42, and decreased until day 130 [32]. Re-exposing the dogs after one and two years did not much increase IgG titers.

Different workers have taken different approaches to attributing specific rickettsiae. In 2010, the U.S. Centers for Disease Control and Prevention (CDC), and consequently state, tribal, and local health agencies, stopped reporting data for particular species in lieu of reporting together all spotted fever group (SFG) rickettsiae [33]. *Rickettsia felis*, and other rickettsiae in its transitional group, are also often grouped with SFG [34]. Typhus group rickettsiae, including *Rickettsia typhi*, do not have a national CDC surveillance case definition, as infections they cause are not nationally notifiable [5]. Some studies have used cross-adsorption protocols to differentiate rickettsial antigens [35,36], but these may bring issues such as requirement for sample volume, expense, and unavailability that make them impractical for large seroprevalence surveys. Four-fold rising convalescent titers may implicate a particular *Rickettsia* agent [27,30], but convalescent samples are often difficult to obtain, only serve in patients experiencing a first, acute exposure to the rickettsial agent, and are not accessible in a cross-sectional survey.

We aimed to interrogate our valuable dataset for particular pathogens, including co-exposure to more than one pathogen, rather than non-specifically reporting all seropositive dogs as reactive to “*Rickettsia”* generically. Our approach was to: 1) use unambiguous data from dogs (those reacting only in a single well or where one pathogen had a much stronger titer than others on the slide) to infer the relative abundance of local rickettsiae, and then 2) use these data to identify spatial patterns in seroprevalence and pathogen distribution across populations. Previous work in Japan was able to differentiate *R. typhi* and *R. japonica* by serology in many clinically ill patients by comparing endpoint titers in acute and convalescent sera. However, they reported limited success because they commonly encountered patients with multiple equal titers [27]. Similarly, in Brazil, a four-fold higher endpoint titer was applied to differentiate *Rickettsia amblyommatis*, *Rickettsia bellii*, *R. felis, R. parkeri, R. rickettsii,* and *Rickettsia rhipicephali* in dogs, with 68% of positive dogs co-seroreacting at similar titers [37]. In our study, only 10% of dogs in our dataset had high titers to more than one *Rickettsia* species. Indeed, when we compared results of analyses based on the conservative “attributable” and total seroreactivity datasets, our inferences from seroprevalence-based spatial analyses remained relatively consistent, albeit with minor differences in levels of statistical significance.

Our PERMANOVA analysis of *individual* dogs indicated that only 5% of the variation in infection patterns was explained by our predefined subregions, with widespread co-reactivity at low titers and broad circulation of these pathogens across the region. This underscores the necessity of examining infection dynamics across population scales (rather than focusing on individual dogs) to better characterize circulating pathogens, and of employing diagnostic assays capable of simultaneously detecting all four common rickettsial pathogens to minimize the risk of misdiagnosis. These findings suggest that dog populations, when multiplex-screened for *Rickettsia* spp., would serve as good sentinel indicators of temporal and spatial epidemiological risk for humans [38].

Dogs with antibodies to *R. rickettsii—*the deadliest of the four pathogens we studied—were the least frequently detected, accounting for 6.2% of attributable seroreactivity. High titers were concentrated in several areas with known RMSF outbreaks, specifically in Mexicali and Reynosa, Mexico [38,39]. Elevated titers were also observed in the neighboring Rio Grande Valley, Texas, although, to our knowledge, no RMSF outbreaks have been documented there. Dogs with high titers were not found in California, aligning with the region’s historically lower incidence of RMSF. Lack of travel histories for three dogs with low *R. rickettsii* titers in California shelters precludes evaluation of source of exposure. Of interest, brown dog ticks removed from the dogs from Reynosa, Mexico, were previously assayed for Rickettsia infection, revealing *Rickettsia amblyommatis*, *Rickettsia parkeri*, and *Candidatus* Rickettsia andeanae [40].

Antibodies to *R. massiliae* were found in all areas of our RoF except Imperial County and were especially prevalent (8.8%) in the west of our RoF. Geographic clustering and a concentration of high titers in Ensenada are consistent with either recent introduction or intense local transmission. First reported in North America in Arizona in 2006 [41], *R. massiliae* has since been documented in our RoF in California [2], Baja California [42], and Texas [43]. As a relatively newly recognized zoonotic pathogen in our RoF, the high prevalence of dog exposure to this agent may reflect an under-recognized human and canine risk. Such abundant *R. massiliae* in western areas also raises concern about possible misdiagnosis of RMSF when relying on cross-reacting serological assays that do not differentiate SFG species.

*Rickettsia typhi*, accounting for 6.8% of attributable exposures, is one of the most prevalent rickettsial infections around the world, but its ecology is incompletely understood. Several pathogens are associated with typhus, including *Rickettsia prowazekii* which causes epidemic typhus, and *R. typhi* which causes murine typhus. Although typically mild in both humans and dogs, typhus caused by *R. typhi* can be severe and even fatal, and has been experiencing a resurgence in parts of our RoF(5, 7). Often associated with warm coastal areas [44], human cases in Mexico are concentrated in the states of Sinaloa, Baja California Sur, and Nuevo León, but also have been reported inland in states like Morelos [4]. In the USA, there are endemic foci in southern California and Texas. In Texas, a “suburban cycle” involving cats and opossums has been proposed in lieu of the classic rat-flea model [6]. In our study, *R. typhi* seroreactivity in dogs conformed with expected coastal patterns but was also found inland in Mexicali and Imperial County, possibly reflecting suitable microclimates or novel transmission cycles. The higher *R. typhi* prevalence among shelter dogs compared with neighborhood dogs was the only demographic difference for any pathogen we detected in this study. This higher prevalence of *R. typhi* among shelter dogs suggests that shelter environments may facilitate exposure to *R. typhi* both for humans and companion animals, consistent with prior studies. Surveys of shelter dogs in Spain and Italy also found evidence of exposure to typhus group rickettsiae [45,46]. Our results highlight the need to include both neighborhood and shelter dogs in surveillance studies to better understand local transmission dynamics and risk heterogeneity across different urban animal populations. Moreover, these findings underscore the importance of implementing flea control measures in shelter environments.

Antibodies to *R. felis* were the most commonly detected (10.9%) in our study. Associated with numerous arthropod vectors including cat fleas (*Ctenocephalides felis)*, *R. felis* infection is thought to be often overlooked due to its mild or nonspecific symptoms in humans [47]. It may be maintained in dogs which can experience prolonged rickettsemia and transmit the pathogen to fleas [12]. Given the high prevalence we observed, further research to understand its interactions with other *Rickettsia* species and enhanced surveillance efforts are warranted.

Cross-protection between closely related *Rickettsia* species has been proposed as a mechanism for pathogen interference and is being explored for vaccine development. *A priori,* one would expect stronger cross-reaction serologically and cross-protection immunologically between more closely related rickettsiae, e.g., among SFG rickettsiae rather than between SFG species and typhus-group species. We found that SFG *R. rickettsii* and *R. massiliae* were negatively correlated in the NMDS and rarely co-detected. This was especially notable in Ensenada, where antibodies to *R. massiliae* were the most prevalent and antibodies to *R. rickettsii* were rarely detected despite numerous reported human RMSF cases. Conversely, in the Rio Grande Valley, *R. rickettsii* predominated while *R. massiliae* was uncommon. These spatial patterns suggest that cross-protective immunity or competitive exclusion may be influencing the distribution of these pathogens. Other factors, including climate and the movement of dogs (and the pathogens they carry), may also influence these spatial patterns. Longitudinal studies could potentially help inform whether *R. massiliae* is expanding in range and/or competing with *R. rickettsii*.

Even though there was geographic overlap in seroreactivity, we did not detect high titers of both *R. rickettsii* and *R. typhi* in the same dog, consistent with potential cross-protection between these two species as well. *Rickettsia typhi* does cross-protect against the SFG *Rickettsia conorii* [15]. In contrast, we did observe high titers to both *R. rickettsii* and *R. felis* in some individuals and prior research documented *R. rickettsii* and *R. felis* co-circulating in the same household [48]. Some dogs with high titers to *R. massiliae* in our study also had high titers to either *R. typhi* or *R. felis.* As a less pathogenic bacteria, the higher overlap may be due to a lower host immune response.

The relationship between *R. felis* and *R. typhi*, relatively distantly related flea-borne pathogens [49], was more complex. Co-seroreactivity was common and similar seroprevalences to both pathogens were observed in some locations including Tijuana and Ensenada. Experimental evidence confirmed co-infection of *R. typhi* and *R. felis* in *C. felis* [50] indicating the potential for ecological overlap and simultaneous transmission. Despite some co-exposure in our dataset, NMDS analysis suggested that individual dogs were statistically less likely than expected to have co-exposure to the pathogens, likely influenced heavily by the high prevalence of *R. felis*. Future studies will the need to explore whether concurrent exposure in dogs reflects shared vector ecology or true co-infection. Molecular studies combining host and vector data will be critical for disentangling these relationships.

This study has several limitations. Our use of samples obtained opportunistically from shelters and field visits may have introduced sampling bias. For example, samples from the BiTeRs program were collected through veterinary clinics, potentially over-representing sick or injured dogs, whereas the clinical status of dogs sampled elsewhere was often unknown. The cross-sectional design of the study, with only a single time point per dog and asynchronous sampling across sites, further complicates interpretation of the data. As with all serological studies, our ability to infer causality, clinical relevance, or timing of infection is limited. Timing in particular can be difficult to interpret due to factors such as variable seroconversion rates and antibody persistence among individual dogs [51]. Applying both threshold-based and magnitude-based analyses helped avoid overinterpretation of titer magnitudes. A small proportion of samples reached the highest dilution tested (1:512), ranging from 1% to 5% depending on the pathogen. Testing was not performed beyond this dilution, which may introduce minor right-censoring. As the geometric mean is more sensitive to such censoring, we used the median titer as our nonparametric summary because it is more robust to extreme values. Additionally, other *Rickettsia* species not included in our assays, such as *R. asembonensis* and *R. parkeri*, are also known to circulate in the region, limiting our ability to capture the full spectrum of circulating rickettsiae. The lack of consistent sample type (whole blood and serum or plasma) may affect antibody detection. Finally, while PCR could provide information on active infections and clarify co-infection patterns, rickettsial DNA is often transient and present at low levels. As a result, a single cross-sectional sample would likely miss many infections, making serology the most practical approach for this study.

Our study provides a foundation for assessing interspecies interactions in *Rickettsia* exposure, offering insights into potential patterns of co-circulation, immunological cross-protection, and assay cross-reactivity. More fundamentally, the seroprevalences documented here stress how integral dogs may be in the ecology of all four of these rickettsial agents. This study provides an important step toward understanding the complex ecology of rickettsial transmission in dogs near the US–Mexico border. Our findings reveal striking geographic variation and underscore the potential for dogs to serve as sentinels for emerging or underrecognized pathogens like *R. massiliae* and *R. felis* and potential hosts for *R. typhi* and *R. felis*. Ultimately, these data can guide more targeted, species-specific surveillance and control strategies for rickettsial disease in both canine and human populations.

## Supporting information

S1 Fig Map of study locations by region. Symbols indicate subregions: triangles – San Diego subregion (Alpine (1), Bonita (2), Boulevard (3), Campo (4), Carlsbad (5), El Cajon (6), San Diego shelter (7)); pentagons – Tijuana metropolitan area (Tijuana (8), Tecate (9), Rosarito (10)); star – Ensenada (11); square – Imperial subregion (12); circle – Mexicali subregion (13); diamonds – Mexico–Texas border subregion (Reynosa (14), Rio Grande Valley (15)). Map created using ArcGIS software. Base layers made with Natural Earth: https://www.naturalearthdata.com/downloads/. Natural Earth data are in the public domain; terms of use: https://www.naturalearthdata.com/about/terms-of-use/.
(TIF)

S1 Table Pairwise patterns of serological titers against rickettsial pathogens in dogs. (XLSX)

S2 Table Symmetric Proportional Co-Seroreactivity Between Rickettsial Pathogen Pairs in Dogs. (XLSX)

S2 Fig NMDS ordination of *Rickettsia* titers in dogs. Non-metric multidimensional scaling (NMDS) was used to visualize variation in serological profiles among dogs (stress = 0.07). Symbols indicate subregions: red circles (○) represent Ensenada; gold upward-pointing triangles (△) represent the NW Baja Peninsula; green plus signs (+) represent San Diego; teal crosses (×) represent Imperial; blue diamonds (◇) represent Mexicali; and pink downward-pointing triangles (▽) represent the Mexico–Texas border subregion.
(TIF)

S3 Table Percentage of dog seroreactive to each pathogen at low (64–128) and high (256–512) titers (95% confidence interval) in each subregion. (XLSX)

S4 Table Distribution of dogs by sex and age among dogs seroreactive to each pathogen. (XLSX)

S5 Table Distribution of dogs by sex and age across categories of seroreactivity. (XLSX)

S6 Table Seroreactivity among dogs in neighborhoods versus shelters in Mexicali and Tijuana, Mexico. (XLSX)
